# Impact of type 2 diabetes mellitus in the utilization and in-hospital outcomes of surgical mitral valve replacement in Spain (2001–2015)

**DOI:** 10.1186/s12933-019-0866-5

**Published:** 2019-05-10

**Authors:** Ana López-de-Andrés, Javier de Miguel-Díez, Nuria Muñoz-Rivas, Valentín Hernández-Barrera, Manuel Méndez-Bailón, José M. de Miguel-Yanes, Rodrigo Jiménez-García

**Affiliations:** 1 Preventive Medicine and Public Health Teaching and Research Unit, Health Sciences Faculty, Rey Juan Carlos University, Avda. de Atenas s/n, 28922 Alcorcón, Madrid Spain; 2 Internal Medicine Department, Hospital Infanta Leonor, Madrid, Spain; 3 Respiratory Department, Hospital General Universitario Gregorio Marañón, Facultad de Medicina, Universidad Complutense de Madrid (UCM), Instituto de Investigación Sanitaria Gregorio Marañón (IiSGM), Madrid, Spain; 4 Internal Medicine Department, Hospital Universitario Clínico San Carlos, Facultad de Medicina, Universidad Complutense de Madrid (UCM), Madrid, Spain; 5 Internal Medicine Department, Hospital General Universitario Gregorio Marañón, Facultad de Medicina, Universidad Complutense de Madrid (UCM), Madrid, Spain

**Keywords:** Type 2 diabetes mellitus, Surgical mitral valve replacement, Hospitalization, In-hospital mortality

## Abstract

**Background:**

The main aims of this study were to examine the incidence and in-hospital outcomes of mechanical and bioprosthetic surgical mitral valve replacement (SMVR) among patients with and without T2DM.

**Methods:**

We performed a retrospective study using the Spanish National Hospital Discharge Database from 2001 to 2015. We included patients with SMVR codified in their discharge report. We grouped admissions by diabetes status. Propensity score matching (PSM) was used to compare outcomes of isolated SMVR.

**Results:**

We identified 42,937 patients (16.41% with T2DM). Incidence rates of mechanical and bioprosthetic SMVR were higher among T2DM patients than among non-T2DM patients. In both groups of patients, the use of bioprosthetic SMVR increased over time. The use of mechanical valves remained stable among T2DM patients. In T2DM and non-T2DM patients with mechanical SMVR, in hospital mortality (IHM) and MACCE decreased significantly (p < 0.001) from 2001 to 2015. T2DM patients had an overall 11.37% IHM, compared with 10.76% among non-T2DM patients (p = 0.176). Regarding MACCE figures were 14.72% vs. 14.22% (p = 0.320) after mechanical SMVR. Total crude IHM were 14.29% for T2DM patients and 15.13% for those without T2DM with bioprosthetic SMVR (p = 0.165) and 18.22 vs. 19.64%, for a MACCE (p = 0.185). Using PSM we found that the IHM and the MACCE of isolated SMVR did not differ significantly between patients with or without T2DM beside the type of valve replacement. Among T2DM patients, those who received bioprosthetic valves had higher IHM (14.29% vs. 11.37%; p = 0.003) and a higher rate of MACCE (18.22% vs. 14.72%; p = 0.001) than T2DM patients with mechanical SMVR.

**Conclusions:**

In Spain from 2001 to 2015, the incidence rates of hospitalization to undergo mechanical or bioprosthetic SMVR were higher among the population suffering T2DM than among the non-T2DM population. In both groups of patients the use of bioprosthetic SMVR increased over time and the use of mechanical valves remained stable in T2DM. T2DM patients have IHM and MACCE after mechanical and bioprosthetic SMVR which are not significantly different to those found among non-diabetic patients. Among T2DM patients, the crude IHM was significantly higher in those who received a bioprosthetic SMVR than those with mechanical SMVR.

**Electronic supplementary material:**

The online version of this article (10.1186/s12933-019-0866-5) contains supplementary material, which is available to authorized users.

## Background

Surgical mitral valve replacement (SMVR) is the recommended treatment for irreparable valve pathologies, and mechanical or bioprosthetic valves have been the mainstream options. However, these preferences may change in the future with the emergence of transcatheter mitral valve replacement [1].

SMVR is more likely to be performed in patients with several comorbidities, including diabetes, [2, 3] and the overall prevalence of diabetes among patients undergoing SMVR has increased over time [4]. In the US, a retrospective study of 1757 patients who underwent SMVR, from 2000 to 2013, found that 19.8% had diabetes [5]. In Canada and in the US, in more than 20,000 patients with mitral valve pathologies, the prevalence of diabetes was double the prevalence in the general population after adjusting for age (10.9% vs. 5.5% in Canada and 13.4% vs. 7.9% in the USA) [6, 7]. Furthermore, the prevalence of diabetes is rising over time among SMVR patients [8].

Previous studies have found that those suffering from diabetes have a higher long-term mortality after SMVR than those not suffering from diabetes [9]. For other valve procedures, such as surgical aortic valve replacement, suffering from type 2 diabetes (T2MD) has been associated with significantly lower in-hospital mortality (IHM) than among those not suffering from type 2 diabetes [10].

The effect of diabetes on perioperative major adverse cardiovascular and cerebrovascular events (MACCE) and IHM after SMVR is not as well defined. Therefore, the aim of the present study was to examine nationwide trends, characteristics and in-hospital outcomes in mechanical and bioprosthetic SMVR among patients with or without T2DM. To accomplish this aim, we used Spanish National Hospital Discharge Database (SNHDD) data from 2001 to 2015. Also, we aimed to identify which factors are associated with suffering from MACCE among patients with or without T2DM after SMVR according to implanted valve type.

## Methods

### Data source

The SNHDD covers over 95% of all hospital admissions in Spain. This database includes up to 14 discharge diagnoses and up to 20 procedures performed during the hospitalization [11]. The ICD-9-CM is used for coding in the SNHDD.

For this retrospective study, we used data from 2001 to 2015.

### Study population

We selected all admissions of patients aged ≥ 40 years with a code for SMVR (ICD-9-CM codes: 35.23 and 35.24).

We defined T2DM patients as those with ICD-9-CM codes 250.x0 and 250.x2 as the primary or as a secondary diagnosis. Those without these codes were classified as non-T2DM patients. We excluded patients with a diagnosis of type 1 diabetes mellitus (ICD-9-CM codes 250.x1 and 250.x3).

### Covariates

Clinical characteristics included information on the overall comorbidities at the time of discharge, which was assessed by calculating the Charlson comorbidity index (CCI) [12]. Calculation of the CCI was performed excluding diabetes as a disease.

Among the procedures, we specifically analyzed if coronary artery bypass graft (CABG), surgical aortic valve replacement (SAVR), surgical procedures on pulmonary and/or tricuspid valves, intra-aortic balloon counter-pulsation, pacemaker implantation and blood transfusions were performed during the hospitalization for SMVR.

We also collected information on the etiology of the mitral valve disease and the presentation.

The ICD-9-CM codes for the diagnoses identified and the procedures conducted during the hospitalization for SMVR used in our investigation are shown in Additional file 1: Table S1.

According to the SNHDD coding procedures “*Weight loss*” is defined as an “*abnormal weight loss over the last months in the opinion of the treating physician*”. There is no specific BMI to define “*Weight loss*”.

We evaluated the mean length of hospital stay (LOHS) and the IHM. IHM was defined by the proportion of patients who died during admission for each year of study.

### End points

The main end points of our investigation were trends in incidence rates of hospitalizations for SMVR and the incidence of MACCE after SMVR. MACCE include IHM, acute myocardial infarction and ischemic stroke, as described by Newman et al. [11] using ICD-9-CM codes.

### Statistical analysis

The analyses were stratified according to the type of valve used (mechanical or bioprosthetic) and for each group of patients (T2DM patients and non-T2DM patients). We considered three time periods, which included 5 consecutive years each (2001–2005; 2006–2010; and 2011–2015).

The incidence rates of admission for SMVR among T2DM patients and non-T2DM patients per 100,000 inhabitants were estimated using the methods described in previous studies [10]. Poisson regression models adjusted by age and sex were used to assess the time trends for study groups providing incidence rate ratios (IRR) with 95% confidence interval as the measure of association.

A descriptive statistical analysis was performed for categorical and continuous variables. Variables are expressed as proportions and as means with standard deviations. Bivariable analysis was performed using the χ^2^ test and ANOVA, as appropriate.

To identify variables associated with MACCE, we constructed six multivariable logistic regression models, one for each type of SMVR (mechanical and bioprosthetic) and for each group of patients (T2DM patients, non-T2DM patients and both). The variables included in the models were those with significant results in the bivariable analysis and those considered relevant in other investigations. Estimates were reported as odds ratios (ORs) with their 95% CIs.

### Sensitivity analysis

To avoid the confounding effect of CABG and other concomitant heart valve surgery on our main study outcome variables (MACCE and IHM) we conducted a sensitivity analysis. To do so we excluded those T2DM who had undergone a CABG or any other concomitant heart valve surgery (isolated SMVR) and used a propensity score matching (PSM) to select a matched non-T2DM for each T2DM patient. PSM was done using multivariable logistic regression for mechanical and bioprosthetic SMVR patients separately. The variables included to estimate the PSM were year of surgery, age and all the comorbidities analyzed (Additional file 1: Table S1).

Matched variables were compared using the McNemar test for categorical variables and t test for paired-samples for continuous variables.

PSM and all statistical analyses were performed with Stata version 10.1 (Stata, College Station, Texas, USA). Statistical significance was set at p < 0.05 (2-tailed).

### Ethical aspects

The study maintained data confidentiality at all times. Given the anonymous and mandatory nature of the database, it was not necessary to obtain informed consent or approval by an ethics committee in accordance with Spanish legislation.

## Results

In our study, we identified a total of 42,937 hospitalizations of patients aged 40 years or older who underwent SMVR in Spain (2001–2015). We identified 35,904 (83.62%) hospitalized patients who underwent mechanical SMVR and 7033 (16.37%) who underwent bioprosthetic SMVR.

### Trends in mechanical SMVR hospitalizations

The proportion of T2DM patients receiving a mechanical valve decreased from 88.2% in 2001–2005 to 73.6% in 2011–2015 (p < 0.001). Equivalent figures for non-T2DM patients were from 90.5 to 77.7% (p < 0.001).

The incidence of mechanical SMVR among T2DM patients remained stable from 2001 to 2015, with values oscillating between 14.26 and 16.45 cases per 100,000 individuals suffering from T2DM. However, among non-T2DM patients, the incidence decreased significantly from 12.5 cases per 100,000 in 2001 to 9.18 cases per 100,000 in 2015 (p < 0.001) (Additional file 2: Figure S1).

The results of the Poisson regression models showed that the incidence of mechanical SMVR was 1.66 times higher among patients with T2DM than among those without T2DM (IRR 1.66; 95% CI 1.61–1.71).

In patients who underwent mechanical SMVR, there was a significant female predominance (65.31% for T2DM and 61.06% for non-T2DM patients). Overall, patients with T2DM were older (67.3; SD = 7.71 years) than patients without diabetes (64.12; SD = 9.59 years; p < 0.05) and were more likely to undergo simultaneous CABG (16.93% vs. 10.02%) and pacemaker implantation (5.34% vs. 4.07%) (Table 1).

**Table 1:** Incidence and sociodemographic and clinical characteristics of patients hospitalized who underwent a mechanical mitral valve replacement in Spain from 2001 to 2015 according to type 2 diabetes status

	T2DM	2001–2005	2006–2010	2011–2015	Total	Trend
Number of procedures (incidence per 100,000 inhabitants)*	Yes	1640 (16.65)	2011 (17.81)	2102 (16.02)	5753 (16.79)	0.153
	No	10,834 (12.14)	9867 (9.81)	9450 (8.85)	30,151 (10.16)	< 0.001
	Both	12,474 (12.59)	11,878 (10.62)	11,552 (9.63)	35,904 (10.85)	< 0.001
Age, mean (SD)*	Yes	66.34 (7.62)	67.59 (7.5)	67.77 (7.91)	67.3 (7.71)	< 0.001
	No	63.27 (9.59)	64.09 (9.57)	65.12 (9.5)	64.12 (9.59)	< 0.001
Female sex, n (%)*	Yes	1147 (69.94)	1329 (66.09)	1281 (60.94)	3757 (65.31)	< 0.001
	No	6722 (62.05)	6049 (61.31)	5639 (59.67)	18,410 (61.06)	0.002
Coronary artery bypass graft, n (%)*	Yes	285 (17.38)	326 (16.21)	363 (17.27)	974 (16.93)	0.564
	No	1046 (9.65)	966 (9.79)	1010 (10.69)	3022 (10.02)	0.032
Surgical aortic valve replacement, n (%)	Yes	470 (28.66)	640 (31.82)	720 (34.25)	1830 (31.81)	0.001
	No	3314 (30.59)	3277 (33.21)	3192 (33.78)	9783 (32.45)	< 0.001
Other valve procedures on pulmonary or tricuspid valves, n (%)	Yes	337 (20.55)	529 (26.31)	620 (29.5)	1486 (25.83)	< 0.001
	No	2358 (21.76)	2623 (26.58)	2739 (28.98)	7720 (25.6)	< 0.001
Intra-aortic balloon counter-pulsation, n (%)	Yes	53 (3.23)	67 (3.33)	79 (3.76)	199 (3.46)	0.633
	No	352 (3.25)	369 (3.74)	347 (3.67)	1068 (3.54)	0.115
Pacemaker implantation, n (%)*	Yes	63 (3.84)	113 (5.62)	131 (6.23)	307 (5.34)	0.004
	No	323 (2.98)	398 (4.03)	506 (5.35)	1227 (4.07)	< 0.001
Blood transfusion, n (%)	Yes	431 (26.28)	442 (21.98)	540 (25.69)	1413 (24.56)	0.004
	No	2575 (23.77)	2268 (22.99)	2371 (25.09)	7214 (23.93)	0.003
Length of hospital stay, mean (SD)*	Yes	24.61 (19.41)	23.78 (20.32)	20.32 (18.39)	22.75 (19.46)	< 0.001
	No	22.76 (20.45)	22.55 (21.68)	20.38 (20.15)	21.95 (20.79)	< 0.001
In-hospital mortality, n (%)	Yes	221 (13.48)	247 (12.28)	186 (8.85)	654 (11.37)	< 0.001
	No	1275 (11.77)	1049 (10.63)	921 (9.75)	3245 (10.76)	< 0.001
MACCE, n (%)	Yes	291 (17.74)	300 (14.92)	256 (12.18)	847 (14.72)	< 0.001
	No	1613 (14.89)	1414 (14.33)	1261 (13.34)	4288 (14.22)	0.007

MACCE include in-hospital all-cause death, acute myocardial infarction or ischemic stroke

*T2DM* type 2 diabetes mellitus

* p < 0.05 for comparisons between T2DM patients and non-T2DM patients

Patient age increased in patients with and without T2DM (mean 66.34 years and 63.27 years, respectively, in 2001–2005 vs. 63.27 and 65.12 years, respectively, in 2011–2015; all p < 0.001). The proportion of female patients decreased significantly in both groups of patients, from 69.94% in 2001–2005 to 60.94% in 2011–2015 among T2DM patients and from 62.05% in 2001–2005 to 59.67% in 2011–2015 among non-T2DM patients.

We found a significant increase in the frequency of SAVR, other valve procedures on pulmonary or tricuspid valves, and pacemaker implantation in patients with and without T2DM over time. However, blood transfusion decreased in both group of patients during the study period (Table 1).

The mean LOHS for patients with and without diabetes undergoing mechanical SMVR was 24.61 and 22.76 days, respectively, in the period from 2001 to 2005, decreasing to 20.32 and 20.38 days, respectively, in 2013–2015 (all p < 0.001). The overall mean LOHS was significantly higher in patients with T2DM (22.75 vs. 21.95 days).

In T2DM patients with mechanical SMVR, IHM and MACCE decreased significantly (all p < 0.001) over time from 13.48 and 17.74%, respectively, in 2001–2005 to 8.85% and 12.18%, respectively, in 2011–2015. Among patients without T2DM, IHM and MACCE also decreased significantly over time (p < 0.001). No differences were found between the two groups of patients for the overall IHM and MACCE (Table 1). Among those who underwent mechanical SMVR, T2DM patients had 11.37% IHM, compared with 10.76% among the non-T2DM patients (p = 0.176). Regarding MACCE we found 14.72% for T2DM and 14.22% for non-T2DM patients (p = 0.320).

Among those who received a mechanical SMVR the rheumatic etiology of the mitral valve insufficiency is coded in under 4% of T2DM patients with no change overtime. Isolated mitral stenosis is found in 11.12% of T2DM patients and in a significantly smaller (p < 0.001) proportion of non-T2DM patients (9.88%). The most common clinical presentation for both groups is mitral stenosis with insufficiency coded in around 16% of patients (Table 2).

**Table 2:** Comorbidities of patients hospitalized who underwent a mechanical mitral valve replacement in Spain from 2001 to 2015 according to type 2 diabetes status

	T2DM	2001–2005	2006–2010	2011–2015	Total	Trend
Mitral stenosis, n (%)*	Yes	208 (12.68)	220 (10.94)	212 (10.09)	640 (11.12)	0.041
	No	1212 (11.19)	999 (10.12)	768 (8.13)	2979 (9.88)	< 0.001
Rheumatic mitral insufficiency, n (%)*	Yes	72 (4.39)	70 (3.48)	83 (3.95)	225 (3.91)	0.368
	No	428 (3.95)	418 (4.24)	475 (5.03)	1321 (4.38)	0.001
Mitral stenosis with insufficiency, n (%)*	Yes	306 (18.66)	352 (17.5)	269 (12.8)	927 (16.11)	< 0.001
	No	1975 (18.23)	1663 (16.85)	1308 (13.84)	4946 (16.4)	< 0.001
Chronic obstructive pulmonary disease, n (%)*	Yes	142 (8.66)	161 (8.01)	176 (8.37)	479 (8.33)	0.774
	No	695 (6.41)	641 (6.5)	600 (6.35)	1936 (6.42)	0.916
Peripheral vascular disease, n (%)*	Yes	49 (2.99)	85 (4.23)	95 (4.52)	229 (3.98)	0.046
	No	236 (2.18)	349 (3.54)	404 (4.28)	989 (3.28)	< 0.001
Acute renal disease, n (%)*	Yes	148 (9.02)	207 (10.29)	306 (14.56)	661 (11.49)	< 0.001
	No	744 (6.87)	1023 (10.37)	1422 (15.05)	3189 (10.58)	< 0.001
Cerebrovascular disease, n (%)*	Yes	94 (5.73)	111 (5.52)	120 (5.71)	325 (5.65)	0.952
	No	468 (4.32)	423 (4.29)	503 (5.32)	1394 (4.62)	< 0.001
Congestive heart failure, n (%)	Yes	351 (21.4)	429 (21.33)	510 (24.26)	1290 (22.42)	0.040
	No	2177 (20.09)	2023 (20.5)	2223 (23.52)	6423 (21.3)	< 0.001
Atrial fibrillation, n (%)*	Yes	969 (59.09)	1238 (61.56)	1298 (61.75)	3505 (60.92)	0.194
	No	6152 (56.78)	5822 (59)	5549 (58.72)	17,523 (58.12)	0.002
Pulmonary hypertension, n (%)*	Yes	513 (31.28)	626 (31.13)	632 (30.07)	1771 (30.78)	0.667
	No	2846 (26.27)	2735 (27.72)	2648 (28.02)	8229 (27.29)	< 0.001
Coronary artery disease, n (%)*	Yes	445 (27.13)	516 (25.66)	571 (27.16)	1532 (26.63)	0.474
	No	1818 (16.78)	1674 (16.97)	1788 (18.92)	5280 (17.51)	< 0.001
Obesity, n (%)*	Yes	157 (9.57)	224 (11.14)	306 (14.56)	687 (11.94)	< 0.001
	No	489 (4.51)	599 (6.07)	719 (7.61)	1807 (5.99)	< 0.001
Cardiogenic shock, n (%)*	Yes	53 (3.23)	65 (3.23)	67 (3.19)	185 (3.22)	0.996
	No	387 (3.57)	414 (4.2)	415 (4.39)	1216 (4.03)	0.008
Gastrointestinal hemorrhage, n (%)	Yes	6 (0.37)	7 (0.35)	3 (0.14)	16 (0.28)	0.333
	No	52 (0.48)	41 (0.42)	34 (0.36)	127 (0.42)	0.417
Endocarditis, n (%)	Yes	106 (6.46)	207 (10.29)	279 (13.27)	592 (10.29)	< 0.001
	No	815 (7.52)	986 (9.99)	1212 (12.83)	3013 (9.99)	< 0.001
Pneumonia, n (%)*	Yes	51 (3.11)	51 (2.54)	29 (1.38)	131 (2.28)	0.001
	No	341 (3.15)	307 (3.11)	208 (2.2)	856 (2.84)	< 0.001
Renal disease, n (%)*	Yes	108 (6.59)	178 (8.85)	270 (12.84)	556 (9.66)	< 0.001
	No	430 (3.97)	529 (5.36)	669 (7.08)	1628 (5.4)	< 0.001
Liver disease, n (%)*	Yes	66 (4.02)	77 (3.83)	106 (5.04)	249 (4.33)	0.124
	No	260 (2.4)	330 (3.34)	430 (4.55)	1020 (3.38)	< 0.001
Cancer, n (%)	Yes	13 (0.79)	23 (1.14)	21 (1)	57 (0.99)	0.566
	No	72 (0.66)	98 (0.99)	99 (1.05)	269 (0.89)	0.007
Weight loss, n (%)	Yes	8 (0.49)	6 (0.3)	13 (0.62)	27 (0.47)	0.321
	No	38 (0.35)	41 (0.42)	39 (0.41)	118 (0.39)	0.699

*T2DM* type 2 diabetes mellitus

* p < 0.05 for comparisons between T2DM patients and non-T2DM patients

The most common associated comorbidities for hospitalized T2DM patients were atrial fibrillation (60.92%), pulmonary hypertension (30.78%) and coronary artery disease (26.63%) (Table 2).

Overall, patients with T2DM had more comorbidities than non-T2DM patients (mean CCI 0.95 ± 0.77 vs. 0.80 ± 0.75, p < 0.05) (Table 2).

We detected a significant increase in comorbidities according to the mean CCI over time in both patients with and without T2DM.

### Trends in bioprosthetic SMVR hospitalizations

Bioprosthetic valves represented 9.8% of all valve replacements in 2001–2005 and increased to 22.4% in 2011–2015 (p < 0.001). Significant increases, from 11.8 to 26.4% and from 9.5 to 22.3%, were observed for T2DM patients and for non-T2DM patients, respectively (p < 0.001, in both cases).

Additional file 3: Figure S2 shows a significant and constant increase in the hospitalization rates over time for T2DM and non-T2DM patients who underwent bioprosthetic SMVR (from 1.69 to 1.11 cases per 100,000 inhabitants, respectively, in 2001 to 6.26 and 3.26 cases per 100,000, respectively, in 2015; p < 0.001).

The Poisson regression models yielded an adjusted IRR for patients with T2DM who underwent bioprosthetic valve replacement of 1.92 (95% CI 1.82–2.05). Therefore, compared to patients without diabetes, the incidence of bioprosthetic SMVR hospitalizations was almost twice as high among T2DM patients.

The mean age increased significantly (p < 0.001) in both group of patients (in T2DM patients from 72.45 years in 2001–2005 to 74.29 in 2011–2015 and in non-T2DM patients from 72.14 to 74.42 years). We found a significant increase in the frequency of other valve procedures on pulmonary or tricuspid valves and blood transfusion in patients with and without T2DM patients over time (Table 3).

**Table 3:** Incidence and sociodemographic and clinical characteristics of patients hospitalized who underwent a bioprosthetic mitral valve replacement in Spain from 2001 to 2015 according to type 2 diabetes status

	T2DM	2001–2005	2006–2010	2011–2015	Total	Trend
Number of procedures (incidence per 100,000 inhabitants)*	Yes	219 (2.22)	449 (3.98)	627 (4.78)	1295 (3.78)	< 0.001
	No	1134 (1.27)	1898 (1.89)	2706 (2.53)	5738 (1.93)	< 0.001
	Both	1353 (1.37)	2347 (2.10)	3333 (2.78)	7033 (2.13)	< 0.001
Age, mean (SD)	Yes	72.45 (5.84)	74.09 (5.52)	74.29 (5.75)	73.91 (5.72)	0.012
	No	72.14 (7.45)	73.17 (7.48)	74.42 (6.81)	73.55 (7.22)	< 0.001
Female sex, n (%)	Yes	129 (58.9)	280 (62.36)	382 (60.93)	791 (61.08)	0.686
	No	664 (58.55)	1098 (57.85)	1576 (58.24)	3338 (58.17)	0.926
Coronary artery bypass graft, n (%)*	Yes	59 (26.94)	112 (24.94)	137 (21.85)	308 (23.78)	0.243
	No	192 (16.93)	320 (16.86)	472 (17.44)	984 (17.15)	0.855
Surgical aortic valve replacement, n (%)	Yes	59 (26.94)	154 (34.3)	219 (34.93)	432 (33.36)	0.085
	No	354 (31.22)	658 (34.67)	995 (36.77)	2007 (34.98)	0.004
Other valve procedures on pulmonary or tricuspid valves, n (%)	Yes	38 (17.35)	110 (24.5)	186 (29.67)	334 (25.79)	0.001
	No	225 (19.84)	519 (27.34)	826 (30.52)	1570 (27.36)	< 0.001
Intra-aortic balloon counter-pulsation, n (%)	Yes	14 (6.39)	40 (8.91)	30 (4.78)	84 (6.49)	0.025
	No	46 (4.06)	110 (5.8)	154 (5.69)	310 (5.4)	0.081
Pacemaker implantation, n (%)	Yes	21 (9.59)	26 (5.79)	43 (6.86)	90 (6.95)	0.192
	No	61 (5.38)	119 (6.27)	193 (7.13)	373 (6.5)	0.117
Blood transfusion, n (%)	Yes	47 (21.46)	110 (24.5)	188 (29.98)	345 (26.64)	0.022
	No	270 (23.81)	459 (24.18)	803 (29.67)	1532 (26.7)	< 0.001
Length of hospital stay, mean (SD)	Yes	26.68 (19.57)	25.09 (22.3)	20.92 (20.68)	23.34 (21.2)	0.007
	No	26.97 (25.33)	25.32 (24.46)	23.35 (22.19)	24.72 (23.64)	0.001
In-hospital mortality, n (%)	Yes	39 (17.81)	70 (15.59)	76 (12.12)	185 (14.29)	0.073
	No	192 (16.93)	312 (16.44)	364 (13.45)	868 (15.13)	0.003
MACCE, n (%)	Yes	50 (22.83)	83 (18.49)	103 (16.43)	236 (18.22)	0.106
	No	250 (22.05)	408 (21.5)	469 (17.33)	1127 (19.64)	< 0.001

MACCE include in-hospital all-cause death, acute myocardial infarction or ischemic stroke

*T2DM* type 2 diabetes mellitus

* p < 0.05 for comparisons between T2DM patients and non-T2DM patients

The use of SAVR increased significantly in non-T2DM patients (31.22% in 2001–2005 vs. 36.77% in 2011–2015; p = 0.004). In T2DM patients, we found a reduction in the use of intra-aortic balloon counter-pulsation over time (6.39% in 2001–2005 vs. 4.78% in 2011–2015; p = 0.025). Over the entire period, T2DM patients were more likely to receive concomitant CABG than non-T2DM patients (23.78% vs. 17.15%; p < 0.001) (Table 3).

In patients with and without T2DM, the mean LOHS decreased significantly over time (26.68 and 26.97 days, respectively, in the first period vs. 20.92 and 23.35 days, respectively, in the last one) (Table 3).

For the total time period, the rates of crude IHM were 14.29% for T2DM patients and 15.13% for those without T2DM who received a bioprosthetic valve (p = 0.165). 18.22%, of T2DM and 19.64%, of non-T2DM suffered a MACCE over the study period (p = 0.185).

In non-T2DM patients, IHM and MACCE decreased significantly over time (p < 0.001). However, no significant changes in frequency were observed in IHM (17.81% in 2001–2005 vs. 12.12% in 2011–2015; p = 0.073) and in MACCE (22.83% in 2001–2005 vs. 16.43% in 2011–2015; p = 0.106) in T2DM patients who received a bioprosthetic valve from 2001 to 2015 (Table 3).

As can be seen in Table 4 for those T2DM patients with a bioprosthetic valve SMVR rheumatic mitral valve insufficiency is found in 3.09%, isolated mitral stenosis in 5.79% and stenosis with insufficiency coded in 10.66% of patients. The proportions found among non-T2DM patients are similar to those found among T2DM patients and no changes are observed overtime in neither group.

**Table 4:** Comorbidities of patients hospitalized who underwent a bioprosthetic mitral valve replacement in Spain from 2001 to 2015 according to type 2 diabetes status

	T2DM	2001–2005	2006–2010	2011–2015	Total	Trend
Mitral stenosis, n (%)	Yes	15 (6.85)	23 (5.12)	37 (5.9)	75 (5.79)	0.660
	No	67 (5.91)	96 (5.06)	109 (4.03)	272 (4.74)	0.032
Rheumatic mitral insufficiency, n (%)	Yes	8 (3.65)	12 (2.67)	20 (3.19)	40 (3.09)	0.773
	No	28 (2.47)	87 (4.58)	82 (3.03)	197 (3.43)	0.002
Mitral stenosis with insufficiency, n (%)	Yes	25 (11.42)	55 (12.25)	58 (9.25)	138 (10.66)	0.268
	No	109 (9.61)	187 (9.85)	238 (8.8)	534 (9.31)	0.442
Charlson Comorbidity Index, mean (SD)	Yes	1.05 (0.88)	0.96 (0.79)	1.12 (0.85)	1.05 (0.83)	0.167
	No	0.87 (0.78)	0.98 (0.8)	1.06 (0.86)	0.99 (0.83)	< 0.001
Chronic obstructive pulmonary disease, n (%)	Yes	27 (12.33)	40 (8.91)	52 (8.29)	119 (9.19)	0.199
	No	81 (7.14)	130 (6.85)	218 (8.06)	429 (7.48)	0.276
Peripheral vascular disease, n (%)	Yes	7 (3.2)	16 (3.56)	32 (5.1)	55 (4.25)	0.326
	No	43 (3.79)	101 (5.32)	142 (5.25)	286 (4.98)	0.119
Acute renal disease, n (%)	Yes	31 (14.16)	80 (17.82)	128 (20.41)	239 (18.46)	0.110
	No	145 (12.79)	318 (16.75)	575 (21.25)	1038 (18.09)	< 0.001
Cerebrovascular disease, n (%)	Yes	11 (5.02)	19 (4.23)	33 (5.26)	63 (4.86)	0.735
	No	47 (4.14)	102 (5.37)	155 (5.73)	304 (5.3)	0.134
Congestive heart failure, n (%)	Yes	64 (29.22)	114 (25.39)	185 (29.51)	363 (28.03)	0.304
	No	287 (25.31)	519 (27.34)	818 (30.23)	1624 (28.3)	0.004
Atrial fibrillation, n (%)	Yes	113 (51.6)	253 (56.35)	377 (60.13)	743 (57.37)	0.077
	No	562 (49.56)	1078 (56.8)	1587 (58.65)	3227 (56.24)	< 0.001
Pulmonary hypertension, n (%)	Yes	66 (30.14)	133 (29.62)	195 (31.1)	394 (30.42)	0.869
	No	271 (23.9)	564 (29.72)	791 (29.23)	1626 (28.34)	0.001
Coronary artery disease, n (%)*	Yes	87 (39.73)	165 (36.75)	229 (36.52)	481 (37.14)	0.684
	No	297 (26.19)	508 (26.77)	727 (26.87)	1532 (26.7)	0.908
Obesity, n (%)*	Yes	26 (11.87)	43 (9.58)	87 (13.88)	156 (12.05)	0.102
	No	39 (3.44)	85 (4.48)	162 (5.99)	286 (4.98)	0.002
Cardiogenic shock, n (%)	Yes	11 (5.02)	22 (4.9)	23 (3.67)	56 (4.32)	0.530
	No	67 (5.91)	106 (5.58)	144 (5.32)	317 (5.52)	0.761
Endocarditis, n (%)	Yes	32 (14.61)	74 (16.48)	123 (19.62)	229 (17.68)	0.176
	No	154 (13.58)	337 (17.76)	618 (22.84)	1109 (19.33)	< 0.001
Pneumonia, n (%)	Yes	6 (2.74)	17 (3.79)	15 (2.39)	38 (2.93)	0.403
	No	59 (5.2)	63 (3.32)	104 (3.84)	226 (3.94)	0.034
Renal disease, n (%)*	Yes	20 (9.13)	45 (10.02)	99 (15.79)	164 (12.66)	0.004
	No	73 (6.44)	153 (8.06)	305 (11.27)	531 (9.25)	< 0.001
Liver disease, n (%)	Yes	7 (3.2)	12 (2.67)	27 (4.31)	46 (3.55)	0.344
	No	39 (3.44)	65 (3.42)	119 (4.4)	223 (3.89)	0.167
Cancer, n (%)	Yes	4 (1.83)	6 (1.34)	4 (0.64)	14 (1.08)	0.278
	No	10 (0.88)	27 (1.42)	38 (1.4)	75 (1.31)	0.371
Weight loss, n (%)	Yes	0 (0)	1 (0.22)	4 (0.64)	5 (0.39)	0.334
	No	3 (0.26)	7 (0.37)	11 (0.41)	21 (0.37)	0.802

*T2DM* type 2 diabetes mellitus

* p < 0.05 for comparisons between T2DM patients and non-T2DM patients

Atrial fibrillation (57.37%), coronary artery disease (37.14%) and pulmonary hypertension (30.42%) were the most common comorbidities for T2DM patients who underwent bioprosthetic valve replacement. Overall, compared to non-T2DM patients, T2DM patients had higher rates of coronary artery disease (37.12% vs. 26.7%), obesity (12.05% vs. 4.98%) and renal disease (12.66% vs. 9.25%) (Table 4).

### Differences between T2DM patients admitted for mechanical versus bioprosthetic SMVR

When we compared T2DM patients who underwent mechanical SMVR with T2DM patients who underwent bioprosthetic SMVR, the mechanical valve replacement patients were younger than the bioprosthetic valve replacement patients (67.3 years vs. 73.91 years; p < 0.001).

For patients with T2DM the prevalence of isolated mitral stenosis (p < 0.001) and mitral stenosis with insufficiency (p < 0.001) is higher among those who underwent a mechanical than a bioprosthetic valve replacement.

T2DM patients who received bioprosthetic versus mechanical valves had more comorbidities (mean CCI, 1.05 ± 0.83 vs. 0.95 ± 0.77; p < 0.001) and required more concomitant CABG (23.78% vs. 16.93%; p = 0.023), intra-aortic balloon counter-pulsation (6.49% vs. 3.46%; p < 0.001), pacemaker implantation (6.95% vs. 5.34%; p = 0.023) and blood transfusion (26.64% vs. 24.56%; p = 0.037).

Among T2DM patients, those who received bioprosthetic valves had higher IHM (14.29% vs. 11.37%; p = 0.003) and a higher rate of MACCE (18.22% vs. 14.72%; p = 0.001) than T2DM patients with mechanical SMVR (Tables 1 and 3).

### Factors associated with MACCE among T2DM patients

The factors independently associated with MACCE according to the type of mitral valve are shown in Tables 5 and 6.

**Table 5:** Multivariable analysis of factors associated with MACCE among type 2 diabetes and non-type 2 diabetes patients who underwent mechanical mitral valve replacement

	T2DM		Non-T2DM		Both	
	OR (95% CI)	p-value	OR (95% CI)	p-value	OR (95% CI)	p-value
Age	1.02 (1.01–1.04)	< 0.001	1.04 (1.03–1.05)	< 0.001	1.04 (1.03–1.05)	< 0.001
Female sex	1.01 (0.85–1.20)	0.882	0.91 (0.84–0.99)	0.013	0.92 (0.86–0.99)	0.028
Coronary artery bypass graft	1.32 (1.07–1.61)	0.008	1.83 (1.65–2.03)	< 0.001	1.69 (1.55–1.85)	< 0.001
Intra-aortic balloon counter-pulsation	7.10 (5.16–9.78)	< 0.001	8.06 (6.96–9.35)	< 0.001	7.88 (6.89–9.01)	< 0.001
Pacemaker implantation	NS	NS	0.62 (0.51–0.76)	< 0.001	0.69 (0.59–0.83)	< 0.001
Blood transfusion	1.34 (1.12–1.59)	0.001	1.49 (1.38–1.62)	< 0.001	1.46 (1.36–1.58)	< 0.001
Peripheral vascular disease	NS	NS	1.33 (1.11–1.60)	0.002	1.29 (1.10–1.52)	0.002
Acute renal disease	4.14 (3.39–5.05)	< 0.001	4.04 (3.68–4.43)	< 0.001	4.07 (3.74–4.42)	< 0.001
Congestive heart failure	1.59 (1.33–1.90)	< 0.001	1.79 (1.65–1.95)	< 0.001	1.76 (1.63–1.89)	< 0.001
Atrial fibrillation	0.57 (0.49–0.67)	< 0.001	0.54 (0.50–0.58)	< 0.001	0.54 (0.51–0.58)	< 0.001
Pulmonary hypertension	NS	NS	0.85 (0.78–0.93)	< 0.001	0.86 (0.80–0.93)	< 0.001
Obesity	NS	NS	0.71 (0.59–0.84)	< 0.001	0.83 (0.72–0.96)	0.011
Gastrointestinal hemorrhage	NS	NS	2.81 (1.88–4.19)	< 0.001	2.52 (1.733.68)	< 0.001
Endocarditis	1.85 (1.47–2.33)	< 0.001	1.64 (1.48–1.83)	< 0.001	1.67 (1.52–1.85)	< 0.001
Pneumonia	2.77 (1.86–4.14)	< 0.001	3.44 (2.93–4.05)	< 0.001	3.35 (2.89–3.90)	< 0.001
Renal disease	1.59 (1.26–2.01)	< 0.001	1.29 (1.12–1.48)	< 0.001	1.34 (1.19–1.51)	< 0.001
Liver disease	3.07 (2.24–4.19)	< 0.001	3.11 (2.66–3.64)	< 0.001	3.13 (2.72–3.60)	< 0.001
Cancer	NS	NS	1.56 (1.14–2.14)	0.006	NS	NS
Weight loss	3.21 (1.36–7.56)	0.008	NS	NS	NS	NS
Year	0.92 (0.90–0.94)	< 0.001	0.95 (0.93–0.95)	< 0.001	0.94 (0.93–0.95)	< 0.001
T2DM	NS	NS	NS	NS	0.96 (0.87–1.05)	0.338

*T2DM* type 2 diabetes mellitus, *NS* not significant

**Table 6:** Multivariable analysis of factors associated with MACCE among type 2 diabetes and non-type 2 diabetes patients who underwent bioprosthetic mitral valve replacement

	T2DM		Non-T2DM		Both	
	OR (95% CI)	p-value	OR (95% CI)	p-value	OR (95% CI)	p-value
Age	1.03 (1.01–1.05)	0.013	1.02 (1.01–1.03)	0.003	1.02 (1.01–1.03)	0.002
Female sex	1.03 (0.73–1.44)	0.677	1.27 (1.08–1.49)	0.009	1.21 (1.04–1.39)	0.008
Coronary artery bypass graft	1.54 (1.07–2.21)	0.020	1.74 (1.45–2.08)	< 0.001	1.71 (1.45–2.00)	< 0.001
Intra-aortic balloon counter-pulsation	9.64 (5.60–16.58)	< 0.001	9.21 (7.01–12.09)	< 0.001	9.46 (7.41–12.06)	< 0.001
Pacemaker implantation	0.39 (0.16–0.90)	0.029	0.46 (0.32–0.67)	< 0.001	0.44 (0.32–0.62)	< 0.001
Blood transfusion	NS	NS	1.36 (1.16–1.60)	< 0.001	1.36 (1.18–1.58)	< 0.001
Acute renal disease	3.67 (2.56–5.28)	< 0.001	3.01 (2.54–3.57)	< 0.001	3.00 (2.57–3.50)	< 0.001
Congestive heart failure	2.30 (1.65–3.22)	< 0.001	1.82 (1.56–2.13)	< 0.001	1.89 (1.64–2.18)	< 0.001
Atrial fibrillation	0.66 (0.48–0.93)	0.017	0.59 (0.50–0.68)	< 0.001	0.59 (0.52–0.68)	< 0.001
Pulmonary hypertension	NS	NS	0.83 (0.70–0.98)	0.031	0.84 (0.72–0.99)	0.034
Obesity	NS	NS	0.54 (0.36–0.82)	0.004	0.66 (0.48–0.91)	0.011
Endocarditis	NS	NS	1.49 (1.25–1.79)	< 0.001	1.49 (1.27–1.75)	< 0.001
Pneumonia	2.91 (1.33–6.37)	0.007	2.96 (2.19–4.03)	< 0.001	3.00 (2.26–3.99)	< 0.001
Renal disease	1.61 (1.05–2.46)	0.029	NS	NS	1.27 (1.04–1.56)	0.021
Liver disease	2.58 (1.29–5.17)	0.007	3.22 (2.36–4.40)	< 0.001	3.09 (2.33–4.11)	< 0.001
Year	0.95 (0.91–0.99)	0.018	0.93 (0.92–0.95)	< 0.001	0.93 (0.92–0.95)	< 0.001
T2DM	NS	NS	NS	NS	0.88 (0.74–1.05)	0.172

*T2DM* type 2 diabetes mellitus, *NS* not significant

Regardless of the type of valve implanted factors that increased MACCE among T2DM patients included older age, concomitant CABG, intra-aortic balloon counter-pulsation during admission, acute or chronic renal disease, congestive heart failure, pneumonia and liver disease.

Blood transfusion during admission, endocarditis, and weight loss increased the risk for MACCE in patients with T2DM with mechanical SMVR. Patients with atrial fibrillation, and pacemaker implantation among those who received a bioprosthetic SMVR, showed lower probability of dying during the hospitalization.

Over time, MACCE decreased significantly regardless of the type of valve used for SMVR among T2DM patients.

### Factors associated with MACCE among non-T2DM patients

As can be seen in Tables 5 and 6 the factor associated with IHM among non-T2DM patients were very similar to those found among diabetic patients.

However, among non-T2DM patients who received a mechanical SMVR, MACCE were significantly higher in those with peripheral vascular disease, in those with gastrointestinal hemorrhage and in those with cancer. For patients who underwent bioprosthetic SMVR, factors that increased MACCE included female sex, blood transfusion during the admission and endocarditis.

As described for T2DM those without the disease also had a significant reduction in both types of valves from 2001 to 2015.

Finally, T2DM did not predict suffering MACCE among patients who underwent mechanical or bioprosthetic mitral valve replacement (OR 0.96; 95% CI 0.87–1.05; p = 0.338 and OR 0.88; 95% CI 0.74–1.05; p = 0.172).

### Sensitivity analysis

Shown in Additional file 4: Table S2 and Additional file 5: Table S3 are the results of the sensitivity analysis. After excluding T2DM patients who had undergone a CABG or any other concomitant heart valve surgery the number of cases were 2232 for mechanical SMVR and 444 for bioprosthetic SMVR. Once PSM was conducted for mechanical SMVR we found that the IHM were 9.77% for T2DM patients and 9.86% for non-T2DM (p = 0.920) and the incidence of MACCE were 13.17% and 13.58% respectively (p = 0.692). For patients with and without T2DM who underwent bioprosthetic SMVR the IHM (13.74% vs. 11.94%; p = 0.422) and the MACCE (17.34% vs. 17.79%; p = 0.860) did not differ significantly between these two groups.

## Discussion

In the past 15 years, there has been an increase in the number of T2DM patients who underwent SMVR in Spain. In our investigation, the prevalence of T2DM among patients who underwent SMVR increased from 13.4 to 16.06% over the study period. Myllykangas et al. [8] described changes in the mitral valve surgery population over 18 years in the Finnish Cardiovascular Diseases Register. These authors found that the prevalence of diabetes in these patients rose from 12.30% in 1997–2002 to 20.9% in 2009–2014 (p < 0.001).

We found that, from 2001 to 2015, the incidence rates of hospitalization to undergo a SMVR were higher among the Spanish population suffering T2DM than the incidence rates for this procedure among the non-T2DM population. These higher incidence rates of hospitalizations were observed for either mechanical or bioprosthetic SMVR. This finding could be due to several factors, such as advanced age and a high index of comorbidities, leading to an increased risk of hospitalization for patients with T2DM and SMVR [9]. In addition, improvements in treatment in terms of short-term and long-term complications have broadened the indication for surgery over the years [13].

Among T2DM patients, we found an increase in the rate of implanted bioprosthetic valves from 2001 to 2015 meanwhile the trend in the use of mechanical valves remained stable. These findings are similar to those reported by the Society of Thoracic Surgeons Adult Cardiac Surgery Database, where the election of mechanical valves has decreased from 68 to 37% during the last decade [2]. Furthermore, a large study based on the Netherlands’ national registry showed that mechanical prostheses are less often implanted, and this occurrence has led to a decrease from 43.4% in 1995 to 13.8% in 2010 [14]. This trend has also been reported in other studies, and these results suggest improved durability of biological prostheses and fewer neurological and functional complications [15]. Although this approach may be reasonable in high-risk and elderly patients, and often occurs in T2DM patients, it is not supported by scientific evidence in younger patients, especially in those without comorbidities or contraindications for anticoagulation [16]. In any case, the selection of the valve type is a decision that should be made by the physician and patient in conjunction and must be individualized. For this decision, the risks for reoperation and for chronic anticoagulation and their consequences, in addition to characteristics of the patient, lifestyle, comorbidities and life expectancy, should be considered in order to increase quality of life and survival [17].

In our opinion factors such as the greater use of bioprosthetic SMVR, increased number of patients who undergo mitral valve plasty/repair (as will be commented in the limitations section) and lastly, technological advances, like the transcatheter mitral valve procedure (TMVP), that have provided new alternatives to surgery in selected patients, could explain the stable trend in the use of mechanical valves from 2001 to 2015 among T2DM patients in our country.

As in patients who underwent SAVR, age in SMVR plays an important role in valve type selection for patients with T2DM [8, 18], with most patients aged > 73 years receiving bioprosthetic valves and patients with a younger age receiving mechanical valves.

IHM following mitral valve replacement is high [3]. We found that the IHM and MACCE for mechanical SMVR have decreased significantly over the last 15 years in both patients with T2DM and without T2DM. However, no significant changes in the IHM and the MACCE have been observed in those patients with diabetes who underwent bioprosthetic SMVR. Berginzi et al. [3] found that IHM of isolated bioprosthetic SMVR improved over time (7.8% in 2003 to 4.7% in 2014, p trend = 0.016), but surgery remained associated with significant morbidity, lengthy hospitalizations and high cost of care. They concluded that these findings highlight the importance of the evolving TMVP field in addressing the critical need to find alternatives with lower morbidity for these patients [3].

The IHM after SMVR found in our investigation is high when compared to studies from other countries [2, 3, 5, 8, 15, 19]. Possible explanations for this higher mortality could include that surgeries are conducted in Spain in patients with higher age and worse baseline conditions. Previous studies have found that the most important determinants of survival after mitral valve replacement are age and comorbidity [2, 3, 5, 8, 15, 19]. Other reasons that may be suggested are differences in the use of concomitant heart procedures and in the health care system characteristics and organization. If we compare our results with those described in Finland, European country with a similar health system to ours, we see that for the period 2009–2014 their 28-day mortality after mitral valve replacement was 8.1% in line with our result for the period 2011–2015 (10.39%) [8]. When we analyze the characteristic of patients who undergo surgery we observe that the mean age is similar in both countries (around 68 years) but the Spanish population has higher prevalence of comorbid conditions and undergo concomitant heart procedures in a greater proportion than the Finnish population (e.g. CABG > 12% in Spain vs. 7.5% in Finland) [8]. Beside these previous comments, the comparison in the mortality rates between countries is complicated due to the differences in data sources, study designs and the study variables collected among others possible reasons.

Our results showed that T2DM patients have rates of mortality and MACCE after mechanical and bioprosthetic SMVR which are not significantly different to those found among non-diabetic patients. This conclusion was confirmed after sensitivity analysis using PSM. One explanation may be that patients who undergo SMVR in the current era have high-risk features, including a significant prevalence of comorbid conditions (e.g., hypertension, anemia, atrial fibrillation, prior sternotomy, coronary artery disease, coagulopathy and chronic renal insufficiency) [3] and not only diabetes.

In our study, SMVR in T2DM patients with a mechanical valve, compared to those with a bioprosthetic valve, was associated with lower IHM and MACCE. In the general population, the choice of biologic or mechanical prosthesis does not significantly affect long-term patient survival after mitral valve replacement [19]. However, a study of 3433 patients (aged 50–69 years) who underwent SMVR in New York state hospitals from 1997 to 2007 found that mechanical prosthetic valves were associated with a lower risk for reoperation but greater risk for bleeding and stroke. The authors concluded that bioprosthetic valves are commonly used in patients judged to have shorter life expectancies based on characteristics such as frailty [20]. This finding could be a possible explanation for our results.

Older age and more comorbidities are factors that increased MACCE in patients with T2DM regardless of the type of valve implanted. These findings are similar to those reported in other studies [14] and in the general population [21, 22].

Diabetes is a predictor of long-term mortality for patients undergoing SMVR [14] and in those with concomitant CABG [23]. In our study, we found significantly higher MACCE in T2DM patients with concomitant CABG and with intra-aortic balloon counter-pulsation independent of the type of valve used than in T2DM patients without these procedures. These data might be useful when interpreting the emerging literature on TMVP [3].

We found that factors associated with a lower risk of dying or suffering MACCE after SMVR included atrial fibrillation, pacemaker implantation, pulmonary hypertension and obesity. The protective effect of atrial fibrillation and pacemaker implantation has been previously found in T2DM patients undergoing surgical aortic valve replacement (SAVR) and atrial fibrillation among patients suffering COPD with SAVR [24, 25]. In our opinion this may be consequence of a selection bias, with patients with arrhythmia undergoing heart surgery having a better baseline comorbidity profile than those without these disorders. This explanation could also be applicable for pulmonary hypertension.

Regarding obesity, several but not all, authors have reported the existence of the “obesity paradox” after cardiac surgery, finding that overweight and obese patients had lower mortality and adverse perioperative outcomes compared with normal weight, underweight, and morbidly obese patients [26–29].

There are some points that should be taken into consideration when interpreting the results of the present study.

In our investigation we analyzed the use of SMVR but not of those patients who underwent mitral valve plasty/repair that is nowadays a common operation in many centers. Therefore the influence of the use of valve plasty/repair in our results is not evaluated. As can be seen in Additional file 6: Table S4 the number of mitral valve plasty/repair has increased significantly over the study period in Spain, from 277 cases in 2001–2005 to 799 cases in 2011–2015 among T2DM patients, and from 1792 to 4010 cases among non-T2DM patients. As can be seen in Additional file 6: Table S4 even if the use of these procedures is increasing overtime, and possibly replacing SMVR, they still represent under 25% of all procedures conducted on mitral valves in Spain for those aged 40 years or over in the period 2011–2015. Further studies are needed in the future to assess the time trends in mitral valve plasty/repair in our country.

We used the SNHDD, an administrative database rather than a clinical database [11]. Therefore, it was subject to coding error. In addition, it is based on mandatory discharge data, and it lacks important clinical details that likely influence treatment selection and outcomes (e.g., ejection fraction, valve size, and medication history).

Unfortunately in the SNHDD among 35,151 patients who received mechanical SMVR only 11,038 (30.74%) had information regarding the etiology of the mitral valve disease and presentation (mitral stenosis, rheumatic mitral insufficiency or mitral stenosis with insufficiency). For those with bioprosthetic SMVR this proportion was even lower 17.86% (1256/7033). The lack of this information makes impossible to analyze these relevant variables and constitutes an important limitation of our investigation.

Despite these limitations, the quality and validity of our dataset has been assessed and has been found to be useful for health research [10]. Furthermore, this dataset reflects the outcome of a real-world population, which is different from that selected in randomized controlled trials. Thus, the results could be extrapolated to the general population.

## Conclusions

In Spain from 2001 to 2015, the incidence rates of hospitalization to undergo mechanical or bioprosthetic SMVR were higher among the population suffering T2DM than among the non-T2DM population. In both groups of patients the use of bioprosthetic SMVR increased over time and the use of mechanical valves remained stable in T2DM. T2DM patients have IHM and MACCE after mechanical and bioprosthetic SMVR which are not significantly different to those found among non-diabetic patients. Among T2DM patients, the crude IHM was significantly higher in those who received a bioprosthetic SMVR than those with mechanical SMVR.

## Additional files

**Additional file 1: Table S1.** Diagnosis and procedures analyzed with their corresponding ICD-9-CM codes.

**Additional file 2: Figure S1.** Incidence of mechanical mitral valve replacement among type 2 diabetes and non-type 2 diabetes patients in Spain 2001–2015.

**Additional file 3: Figure S2.** Incidence of bioprosthetic mitral valve replacement among type 2 diabetes and non-type 2 diabetes patients in Spain 2001–2015.

**Additional file 4: Table S2.** Distribution according to study variables of propensity score–matched T2DM and non-T2DM patients who underwent a mechanical surgical mitral valve replacement.

**Additional file 5: Table S3.** Distribution according to study variables of propensity score–matched T2DM and non-T2DM patients who underwent a bioprosthetic surgical mitral valve replacement.

**Additional file 6: Table S4.** Mitral valve plasty/repair hospitalizations in Spain from 2001 to 2015.

## Data Availability

According to the contract signed with the Spanish Ministry of Health and Social Services, which provided access to the databases from the Spanish National Hospital Database (*Conjunto Mínimo Basico de Datos*; CMBD), we cannot share the databases with any other investigator, and we have to destroy the databases once the investigation has concluded. Consequently, we cannot upload the databases to any public repository. However, any investigator can apply for access to the databases by filling out the questionnaire available at http://www.msssi.gob.es/estadEstudios/estadisticas/estadisticas/estMinisterio/SolicitudCMBDdocs/Formulario_Peticion_Datos_CMBD.pdf. All other relevant data are included in the paper.

## Abbreviations

CABG: coronary artery bypass graft
CCI: Charlson comorbidity index
ICD-9-CM: International Classification of Diseases, Ninth Revision, Clinical Modification
IHM: in-hospital mortality
LOHS: length of hospital stay
MACCE: major adverse cardiovascular and cerebrovascular events
PSM: propensity score matching
SAVR: surgical aortic valve replacement
SMVR: surgical mitral valve replacement
SNHDD: Spanish National Hospital Discharge Database
T2DM: type 2 diabetes mellitus
TMVP: transcatheter mitral valve procedure
