# Supplementary material for: Impact of type 2 diabetes mellitus in the utilization and in-hospital outcomes of surgical mitral valve replacement in Spain (2001–2015)
Source: Cardiovasc Diabetol. 2019 May 10;18:60. doi: 10.1186/s12933-019-0866-5 (PMC6511144; doi:10.1186/s12933-019-0866-5)
Supplement: Supplementary file 1 — Additional file 1: Table S1. Diagnosis and procedures analyzed with their corresponding ICD-9-CM codes. [file 12933_2019_866_MOESM1_ESM.docx]

Table S1. Diagnosis and procedures analyzed with their corresponding ICD-9-CM codes.

|  | **ICD-9-CM codes** |
| --- | --- |
| Mitral stenosis | 394.0 |
| Rheumatic mitral insufficiency, | 394.1 |
| Mitral stenosis with insufficiency, | 394.2 |
| Chronic obstructive pulmonary disease | 490, 491, 491.0, 491.1, 491.2x, 491.8, 491.9, 492, 492.0, 492.8, 496 |
| Type 2 diabetes mellitus | 250.x0 and 250.x2 |
| Peripheral vascular disease | 0.93.0,473.3,440.x,441.x,443.1-443.9,447.1,557.1,557.9,V43.4 |
| Renal disease | 403.01, 403.11, 403.91, 404.02, 404.03, 404.12, 404.13, 404.92, 404.93, 582, 583.0–583.7, 585, 586, 588.0, V42.0, V45.1, V56 |
| Cerebrovascular disease | 362.34, 430.x–438.x |
| Congestive heart failure | 398.91,402.01,402.11,402.91,404.01, 404.03,404.11,404.13,404.91,404.93,425.4–425.9, 428.x |
| Ischemic stroke | 433.xx, 434,xx, 436 |
| Atrial fibrillation | 427.31 |
| Pulmonary hypertension | 416.0 and 416.8 |
| Coronary artery disease | 410.xx, 412.x, 413.x, 414.0, 414, 414.00, 414.01, 414.2-9 |
| Acute myocardial infarction | 410.xx |
| Obesity | 278.0 |
| Cardiogenic shock | 785.51 |
| Gastrointestinal hemorrhage | 578, 578.0, 578.1, 578.9 |
| Endocarditis | 424.90, 421.9, 424.1, 421.0 |
| Pneumonia | 480–488, 507.0–507.8 |
| Acute renal disease | 584, 584.5-584.9 |
| Liver disease | 070.22, 070.23, 070.32, 070.33, 070.44, 070.54, 070.6, 070.9, 456.0–456.2, 570.x, 571.x, 572.2–572.8, 573.3, 573.4, 573.8, 573.9, V42.7 |
| Cancer | 140.x–172.x, 174.x–195.x, 196.x–199.x |
| Weight loss | 260, 261, 262, 263.0-263.2, 263.8, 263.9, 783.2, 977.4 |
| Coronary artery bypass graft | 36.10-36.19 |
| Surgical aortic valve replacement | 35.21, 35.22 |
| Other valves procedures on pulmonary or tricuspid valves | 35.33, 35.03, 35.04, 35.13, 35.14, 35.25, 35.26, 35.27, 35.28, 35.33 |
| Intra-aortic ballon counterpulsation | 37.61 |
| Pacemaker implantation | 37.70-37.74; 37.80-37.83 |
| Blood transfusion | 99.00, 99.01-99.08 |
