# Supplementary material for: Impact of type 2 diabetes mellitus in the utilization and in-hospital outcomes of surgical mitral valve replacement in Spain (2001–2015)
Source: Cardiovasc Diabetol. 2019 May 10;18:60. doi: 10.1186/s12933-019-0866-5 (PMC6511144; doi:10.1186/s12933-019-0866-5)
Supplement: Supplementary file 2 — Additional file 2: Figure S1. Incidence of mechanical mitral valve replacement among type 2 diabetes and non-type 2 diabetes patients in Spain 2001–2015. [file 12933_2019_866_MOESM2_ESM.docx]

Figure S1. Incidence of mechanical mitral valve replacement among type 2 diabetes and non-type 2 diabetes patients in Spain 2001-2015
