# Supplementary material for: Impact of type 2 diabetes mellitus in the utilization and in-hospital outcomes of surgical mitral valve replacement in Spain (2001–2015)
Source: Cardiovasc Diabetol. 2019 May 10;18:60. doi: 10.1186/s12933-019-0866-5 (PMC6511144; doi:10.1186/s12933-019-0866-5)
Supplement: Supplementary file 4 — Additional file 4: Table S2. Distribution according to study variables of propensity score–matched T2DM and non-T2DM patients who underwent a mechanical surgical mitral valve replacement. [file 12933_2019_866_MOESM4_ESM.docx]

Table S2. Distribution according to study variables of propensity score–matched T2DM and non-T2DM patients who underwent a mechanical surgical mitral valve replacement.

|  | | T2DM (2232) | Matched Non T2DM (2232) | p |
| --- | --- | --- | --- | --- |
| Time period | 2001/05 | 709(31.77) | 724(32.44) | 0.318 |
|  | 2006/10 | 781(34.99) | 734(32.89) |  |
|  | 2011/15 | 742(33.24) | 774(34.68) |  |
| Female | | 1518(68.01) | 1552(69.53) | 0.272 |
| Age in years, mean (SD) | | 67.88(7.85) | 67.12(8.44) | 0.965 |
| Charlson Comorbidity Index, mean(SD) | | 185(8.29) | 155(6.94) | 0.091 |
| Chronic obstructive pulmonary disease, n(%) | | 64(2.87) | 56(2.51) | 0.459 |
| Peripheral vascular disease, n(%) | | 215(9.63) | 178(7.97) | 0.051 |
| Acute renal disease, n(%) | | 134(6) | 113(5.06) | 0.169 |
| Cerebrovascular disease, n(%) | | 503(22.54) | 473(21.19) | 0.277 |
| Congestive heart failure, n(%) | | 1380(61.83) | 1437(64.38) | 0.077 |
| Atrial fibrillation, n(%) | | 637(28.54) | 652(29.21) | 0.620 |
| Pulmonary hypertension, n(%) | | 355(15.91) | 324(14.52) | 0.196 |
| Coronary artery disease, n(%) | | 294(13.17) | 274(12.28) | 0.369 |
| Obesity, n(%) | | 59(2.64) | 41(1.84) | 0.069 |
| Cardiogenic shock, n(%) | | 9(0.4) | 8(0.36) | 0.808 |
| Endocarditis, n(%) | | 200(8.96) | 176(7.89) | 0.196 |
| Pneumonia, n(%) | | 50(2.24) | 32(1.43) | 0.045 |
| Renal disease, n(%) | | 206(9.23) | 185(8.29) | 0.266 |
| Liver disease, n(%) | | 79(3.54) | 63(2.82) | 0.172 |
| Cancer, n (%) | | 23(1.03) | 21(0.94) | 0.762 |
| Weight loss, n(%) | | 9(0.4) | 5(0.22) | 0.284 |
| Intra-aortic balloon counter-pulsation, n(%) | | 65(2.91) | 58(2.6) | 0.522 |
| Pacemaker implantation, n(%) | | 83(3.72) | 87(3.9) | 0.754 |
| Blood transfusion, n(%) | | 481(21.55) | 479(21.46) | 0.942 |
| Length of hospital stay, mean(SD) | | 21.94(19.25) | 21.23(17.83) | 0.853 |
| In-hospital mortality, n(%) | | 218(9.77) | 220(9.86) | 0.920 |
| MACCE, n(%) | | 294(13.17) | 303(13.58) | 0.692 |

T2DM: Type 2 diabetes mellitus. MACCE include in-hospital all-cause death, acute myocardial infarction or ischemic stroke
