# Supplementary material for: Impact of type 2 diabetes mellitus in the utilization and in-hospital outcomes of surgical mitral valve replacement in Spain (2001–2015)
Source: Cardiovasc Diabetol. 2019 May 10;18:60. doi: 10.1186/s12933-019-0866-5 (PMC6511144; doi:10.1186/s12933-019-0866-5)
Supplement: Supplementary file 5 — Additional file 5: Table S3. Distribution according to study variables of propensity score–matched T2DM and non-T2DM patients who underwent a bioprosthetic surgical mitral valve replacement. [file 12933_2019_866_MOESM5_ESM.docx]

Table S3. Distribution according to study variables of propensity score–matched T2DM and non-T2DM patients who underwent a bioprosthetic surgical mitral valve replacement.

|  | | T2DM (444) | Matched Non T2DM (444) | p |
| --- | --- | --- | --- | --- |
| Time period | 2001/05 | 84(18.92) | 90(20.27) | 0.781 |
|  | 2006/10 | 157(35.36) | 148(33.33) |  |
|  | 2011/15 | 203(45.72) | 206(46.4) |  |
| Female | | 272(61.26) | 283(63.74) | 0.446 |
| Age in years, mean (SD) | | 73.15(6.34) | 73.14(6.72) | 0.984 |
| Charlson Comorbidity Index, mean(SD) | | 1.04(0.94) | 1.07(0.96) | 0.647 |
| Chronic obstructive pulmonary disease, n(%) | | 46(10.36) | 40(9.01) | 0.496 |
| Peripheral vascular disease, n(%) | | 20(4.5) | 13(2.93) | 0.214 |
| Acute renal disease, n(%) | | 77(17.34) | 79(17.79) | 0.860 |
| Cerebrovascular disease, n(%) | | 26(5.86) | 26(5.86) | 0.999 |
| Congestive heart failure, n(%) | | 125(28.15) | 131(29.5) | 0.657 |
| Atrial fibrillation, n(%) | | 252(56.76) | 267(60.14) | 0.307 |
| Pulmonary hypertension, n(%) | | 124(27.93) | 133(29.95) | 0.505 |
| Coronary artery disease, n(%) | | 98(22.07) | 86(19.37) | 0.320 |
| Obesity, n(%) | | 64(14.41) | 64(14.41) | 0.999 |
| Cardiogenic shock, n(%) | | 17(3.83) | 19(4.28) | 0.734 |
| Endocarditis, n(%) | | 93(20.95) | 96(21.62) | 0.806 |
| Pneumonia, n(%) | | 11(2.48) | 15(3.38) | 0.426 |
| Renal disease, n(%) | | 57(12.84) | 52(11.71) | 0.609 |
| Liver disease, n(%) | | 15(3.38) | 14(3.15) | 0.850 |
| Cancer, n (%) | | 3(0.68) | 2(0.45) | 0.654^a^ |
| Weight loss, n(%) | | 2(0.45) | 3(0.68) | 0.654^a^ |
| Intra-aortic balloon counter-pulsation, n(%) | | 28(6.31) | 19(4.28) | 0.177 |
| Pacemaker implantation, n(%) | | 21(4.73) | 21(4.73) | 0.999 |
| Blood transfusion, n(%) | | 98(22.07) | 116(26.13) | 0.158 |
| Length of hospital stay, mean(SD) | | 23.52(23.14) | 23.1(20.33) | 0.772 |
| In-hospital mortality, n(%) | | 61(13.74) | 53(11.94) | 0.422 |
| MACCE n(%) | | 77(17.34) | 79(17.79) | 0.860 |

T2DM: Type 2 diabetes mellitus. MACCE include in-hospital all-cause death, acute myocardial infarction or ischemic stroke
