# Supplementary material for: Impact of type 2 diabetes mellitus in the utilization and in-hospital outcomes of surgical mitral valve replacement in Spain (2001–2015)
Source: Cardiovasc Diabetol. 2019 May 10;18:60. doi: 10.1186/s12933-019-0866-5 (PMC6511144; doi:10.1186/s12933-019-0866-5)
Supplement: Supplementary file 6 — Additional file 6: Table S4. Mitral valve plasty/repair hospitalizations in Spain from 2001 to 2015. [file 12933_2019_866_MOESM6_ESM.docx]

Table S4. Mitral valve plasty/repair hospitalizations in Spain from 2001 to 2015

|  | Time periods | | |  | |
| --- | --- | --- | --- | --- | --- |
|  | 2001-5 | 2006-10 | 2011-15 | Total | Time trend |
| Non T2DM, n (%) | 1792 (13.02) | 2908 (19.82) | 4010 (24.81) | 8710 (19.53) | <0.001 |
| T2DM, n (%) | 277 (12.98) | 545 (18.14) | 799 (22.65) | 1621 (18.69) | <0.001 |
| Total, n (%) | 2069 (13.02) | 3453 (19.53) | 4809 (24.42) | 10331 (19.34) | <0.001 |

ICD9 CM codes to identify mitral valve plasty/repair included 35.02 (Closed heart valvotomy, mitral valve), 35.12 (Open heart valvuloplasty of mitral valve without replacement) and 35.33(Annuloplasty) For Annuloplasty also a code for mitral disease (394.x or 424.0) had to be coded in the same patient
